# Growth of extremely low birth weight infants at a tertiary hospital in a middle-income country

**DOI:** 10.1186/s12887-019-1568-6

**Published:** 2019-07-11

**Authors:** Tendai Mabhandi, Tanusha Ramdin, Daynia Elizabeth Ballot

**Affiliations:** Johannesburg, South Africa

**Keywords:** Extremely low birth weight, Neonate, Growth, Nutrition

## Abstract

**Background:**

Survival of extremely low birth weight (ELBW; birth weight less than 1000 g) infants has improved significantly since the 1990s. Consequently, growth monitoring in ELBW infants has gained more relevance.

**Methods:**

We conducted this study to describe the growth of ELBW infants at a tertiary hospital, to audit macronutrient intake and explore the association of prematurity complications with growth. This was a retrospective study on 92 ELBW infants born at Charlotte Maxeke Johannesburg Academic Hospital. The association between good growth (regaining birth weight in 21 days or less and subsequent growth velocity > 15 g/kg/day) and complications of prematurity was explored.

**Results:**

Only 11infants (13%) had a discharge weight above the 10th centile when the Fenton growth chart was used compared to 20 infants (22.4%) when the Intergrowth 21st Project growth standard was used. The mean weight velocity was 13.5 g/kg/day and the mean number of days to regain birth weight was 18.2 days. Factors associated with poor growth were late-onset sepsis, persistent patent ductus arteriosus, continuous positive airway pressure for more than 2 days, invasive ventilation, oxygen on day 28 and being kept nil per os. Protein and caloric intake correlate positively with growth velocity. Unlike the Fenton Growth Charts, use of the Intergrowth 21st Project growth standards revealed the association between neonatal factors and poor growth.

**Conclusion:**

Growth outcome in infants is poor at 36 weeks postmenstrual age at our institution. Intergrowth 21st Project growth standards were superior to Fenton Growth Charts, however a multicentre study is required before adoption.

## Background

Survival of extremely low birth weight (ELBW) infants has improved significantly since the 1990s owing to advances in obstetric and neonatal care [1]. Consequently, growth monitoring of ELBW infants has gained more interest. Poor growth among ELBW infants has been well documented [2]. Although ELBW infants have compensatory growth into their early adult years, they remain shorter than their predicted mid-parental heights [3]. Low birth weight infants have also been noted to have a poor neurocognitive outcome [4]. There is growing evidence among ELBW infants that inadequate nutrition during the early weeks of life leads to persistent growth faltering that may lead to permanent detrimental effects [5]. A study on 148 ELBW infants showed that increased protein and caloric intake in the first week of life was associated with a better neurodevelopmental outcome at 18 months [6].

Nutrition in ELBW infants is surrounded by several challenges. In a low resource setting, ITN (Intravenous Total Nutrition) may not be readily available due to cost limitations [7]. Furthermore, difficult venous access, suspected or established diagnosis of necrotizing enterocolitis (NEC), sepsis and metabolic derangements frequently interfere with ITN and enteral feeds administration [8, 9].

There is little information in South Africa on the growth of ELBW infants and their caloric intake while in hospital. A previous prospective study on very low birth weight (VLBW) infants admitted at Charlotte Maxeke Johannesburg Academic Hospital (CMJAH) showed that infants had initial poor growth followed by catch-up growth but with persistent deficits at 20 months [10]. Another study at CMJAH concluded that VLBW infants had a weight velocity approaching recommended values [11]. A study conducted in Cape Town on ELBW infants showed that weight velocity was approaching the generally accepted standard [12], however, weight Z-score and macronutrient intake were not described in the study. Therefore, the present study aimed to review growth and audit macronutrient intake of ELBW infants in the setting of a tertiary hospital.

## Methods

### Aim

This was a pilot study to review the growth of ELBW infants at CMJAH from birth to 36 weeks postmenstrual age, audit their macronutrient intake and explore the association of prematurity complications with growth.

### Study design

The study was a retrospective longitudinal description of growth and macronutrient intake in ELBW infants at CMJAH.

### Participant characteristics

#### Inclusion criteria

All ELBW infants admitted at CMJAH within 72 h of birth between 01 January 2015 and 31 March 2017 were included in the study.

#### Exclusion criteria

Infants who were transferred to other hospitals or demised before discharge, those with chromosomal abnormalities and major congenital abnormalities were excluded.

### Study process

The study was a secondary analysis of an existing database managed using Research Electronic Data Capture (REDCAP), hosted by the University of Witwatersrand [13] and a review of relevant hospital records. Weight was captured weekly until discharge. The volume of feeds, intravenous (IV) fluids and ITN was captured daily for the first 28 days of life. The number of days to regain birth weight was captured and the mean weight velocity was calculated from the day birth weight was regained to 36 weeks postmenstrual age. Mean weight velocity was calculated using the exponential method [14] as shown below;

$$ \mathrm{Weight}\ \mathrm{velocity}=1000\ \mathrm{X}\ \ln\ \left({\mathrm{Weight}}^{36\ \mathrm{weeks}}/{\mathrm{Weight}}^{\mathrm{Birth}\ \mathrm{weight}\ \mathrm{regained}}\right)/\mathrm{Time}\ \mathrm{interval} $$

The Fenton’s Growth Chart [15] and Intergrowth 21st Project growth standards [16, 17] were used as reference for normal growth. For the Intergrowth 21st Project growth standards, the Intergrowth 21st Project newborn charts were used to derive the birth weight Z-scores and the Intergrowth 21st Project postnatal growth charts were used to derive the Z-scores at 36 weeks. An infant born with a birth weight below the 10th centile was referred to as small for gestational age (SGA) [18]. The delta Z-score from birth to 36 weeks postmenstrual age was determined for each infant. To avoid under-representation of infants with good growth, infants discharged before 36 weeks postmenstrual age had their discharge weight Z-score analyzed as that at 36 weeks. Delta Z-score was calculated using the formula below;

$$ \mathrm{Delta}\ \mathrm{Z}-\mathrm{score}=\mathrm{Weight}\ \mathrm{Z}-\mathrm{score}\ \mathrm{at}\ 36\;\mathrm{weeks}-\mathrm{Weight}\ \mathrm{Z}-\mathrm{score}\ \mathrm{at}\ \mathrm{birth} $$

For the purpose of computation of macronutrients, the nutritional content of the various types of feeds, IV fluids and ITN was derived from product inserts and previous studies (Table 1) [19]. Daily caloric intake in kcal/kg/day was calculated for each infant during the first 28 days for life using the formula below, where volume is in milliliters (mls), weight in grams and the figure 1000 converts grams to kilograms;

$$ \mathrm{Daily}\kern0.5em \mathrm{caloric}\kern0.5em \mathrm{intake}=\kern0.5em \frac{1000\kern0.5em \mathrm{X}\kern0.5em {\mathrm{Volume}}^{\mathrm{Fluid}/\mathrm{ITN}/\mathrm{Feed}\kern0.5em }\mathrm{X}\kern0.5em \mathrm{Calories}\kern0.5em \mathrm{per}\kern0.5em {\mathrm{ml}}^{\mathrm{Fluid}/\mathrm{ITN}/\mathrm{Feed}}}{\mathrm{Current}\kern0.5em \mathrm{heaviest}\kern0.5em \mathrm{weight}}\kern0.5em $$

**Table 1:** Daily infant nutritional requirements and nutritional composition of feeds and parenteral nutrition

	Daily infant Requirement (per kg weight)	Neonatolyte (per dL)	ITN 101 per dL	ITN 102 per dL	ITN 105 per dL	Breastmilk per dL	Prenan per dL	Breastmilk Fortifier (FM85) (per 1 g)
Energy (kcal)	105–130	40	38.3	62.4	46.2	67	80	4.4
Dextrose (g)	–	10	9.57	10.41	6.3	–	–	–
Protein (g)	3.5–4.5 g	0	1.91	2.08	2.1	1.0	2.3	0.4
Lipid (g)	5 – 7 g	0	0	2.08	2.1	3.5	4.2	0.2

Mean weekly caloric intake for each week and mean caloric intake over 28 days was calculated. A mean caloric intake of at least 110 kcal/kg/day was classified as good caloric intake. Daily protein and lipid intake were calculated using a similar formula to the daily caloric intake.

Definitions of complications of prematurity were based on those used in the Vermont Oxford Network (www.vtoxford.org). The need for ventilation (invasive and non-invasive), as well as complications of prematurity including NEC, retinopathy of prematurity (ROP), late onset sepsis (> 72 h after birth), oxygen on day 28 of life and patent ductus arteriosus (PDA) were recorded. Necrotizing enterocolitis and PDA were defined in this study as follows;

- NEC was defined as NEC grade 2 or 3 according to modified Bells’ staging [20]
- ROP was defined as stage 2 or more [21]
- PDA was defined as a hemodynamically significant PDA on echocardiography [22]

### CMJAH neonatal unit

The unit’s feeding protocol was as follows: Prescription of feeds was at the discretion of the attending physician. Although exclusive breastfeeding was unit protocol, no donor breast milk was available. Hence, the infant formula Prenan (Nestle) was provided for those ELBW infants whose mothers were unwilling or unable to breastfeed. Newly born infants were started on intravenous fluids (usually Non-K Neonatolyte) at 80 to 100mls/kg/day on the first day of life. Feeds were introduced on the second day of life starting at 20mls/kg/day. Feeds were gradually increased by 20 to 30mls/kg/day replacing intravenous fluids until 160 to180mls/kg/day of full enteral feeds was reached. Upon reaching full enteral feeds, each feed of breastmilk was fortified with 1 g FM85 (Nestle). Enteral feeds were discontinued if there was evidence of feeding intolerance. Infants who were not on full feeds for more than 48 h due to any reason had ITN prescribed for them provided no contraindications to ITN administration were present. Infants were weighed twice a week on an electronic scale (NAGATA BW-20, Taiwan). In the absence of ongoing complications requiring hospital care, infants were discharged upon attaining 1600 g. Infants with respiratory distress syndrome (RDS) were offered nasal continuous positive airway pressure (NCPAP) and surfactant. Upon delivery, infants with RDS were transferred to a Transitional Unit where they were offered NCPAP if available. This was done while the infant awaited transfer to their final destination, the Premature Unit, where NCPAP was usually available.

### Statistical analysis

Data was entered into a Microsoft Excel Spreadsheet for data cleaning. The final dataset was exported into IBM SPSS 25 for analysis. The distribution of continuous variables was explored. Those variables with a normal distribution were described using mean and standard deviation (SD) while those with a skewed distribution were described using median and interquartile range (IQR). Categorical variables were described by frequencies and percentages.

An infant with good growth was defined as that infant who regained birth weight in 21 days or less and had a weight velocity of at least 15 g/kg/day [23, 24]. An infant with poor growth was defined as that infant who regained birth weight after 21 days and/or had a growth velocity less than 15 g/kg/day. The association of growth with complications of prematurity was assessed in three ways as follows;

- Comparison of occurrence of prematurity complications between infants with good and those with poor growth.
- Comparison of the occurrence of prematurity complications between infants who attained a weight above 10th centile at 36 weeks and those who failed to attain such a weight.
- Delta Z-scores from birth to 36 weeks were analyzed as a continuous variable.

For continuous variables, student t-test was used for variables with normal distribution and Mann Whitney U-test for ranked data or those variables with a skewed distribution. The Chi-squared test of independence was used for categorical variables.

## Results

### Sample characteristics

There were 2829 infants with birth weight less than 1500 g infants captured on the database. Of these, 886 were ELBW infants. After excluding those outside the study period, 325 infants remained. Ten infants were transferred to other hospitals and 181 infants died in hospital. Therefore, 134 infants were eligible for the study. Forty-two infants had missing records leaving a final sample of 92. For derivation of weight Z-scores and centiles, 89 were included because 3 infants had a gestational age less than 24 weeks and therefore could not be plotted on the Intergrowth 21st Project newborn charts. Characteristics of the sampled infants are displayed in Table 2.

**Table 2:** Characteristics of sample population

	*N* = 92
Baseline characteristics	
Females, frequency (percentage)	47 (51.1%)
Birth weight in grams, mean (SD)	867 (81.4)
Birth weight Z -score, mean (SD)	0.4 (1.1)
Gestational age at delivery in weeks, mean (SD)	27.6 (2.0)
Duration of hospital stay in days, mean (SD)	69.1 (17.1)
Postmenstrual age at discharge in weeks, mean (SD)	36.7 (2.7)
Kangaroo care, frequency (percentage)	46 (50.0%)
Predominantly breastfed at discharge	49 (52.3%)
Prenatal factors	
Chorioamnionitis, frequency (percentage)	2 (2.2%)
Antenatal steroids, frequency (percentage)	51 (55.4%)
Postnatal complications	
Patent dictus arteriosus, frequency (percentage)	13 (14.1%)
Sepsis after day 3 of life, frequency (percentage)	53 (57.6%)
Invasive ventilation, frequency (percentage)	17 (18.5%)
Continuous positive airway pressure for more than 2 days, frequency (percentage)	29 (31.5%)
Nil per os (excluding the first day of life), frequency (percentage)	61 (66.3%)
Retinopathy of prematurity, frequency (percentage)	79 (85.7%)
Oxygen on day 28 of life, frequency (percentage)	68 (73.9%)
Postnatal steroids, frequency (percentage)	49 (53.3%)
Necrotizing enterocolitis	7 (7.6%)
Surgery for necrotizing enterocolitis, frequency (percentage)	1 (1.1%)
Other surgery	4 (4.3%)

### Growth

The mean birth weight was 867 g (SD = 81.4) with a mean birth weight Z-score of −0.45 (SD = 1.01) and − 0.72 (SD = 1.20) as per Fenton Growth Chart and Intergrowth 21st Project newborn weight charts respectively. Table 3 displays the mean birth weight Z-score, weight Z-score at 36 weeks and delta Z-score as per the Fenton Growth Chart and Intergrowth 21st Project growth standard. The mean percentage weight loss was 7.38% (SD = 5.82) and the mean number of days to regain birth weight was 18.2 days (SD = 10.2). Mean weight velocity was 13.5 g/kg/day (SD = 3.1) for the full length of hospital stay. At 36 weeks postmenstrual age, the mean weight for males was 1494 g (SD = 297) with a mean weight Z-score of − 2.81 (SD = 1.16) and − 3.18 (SD = 1.84) as per Fenton Growth Chart and Intergrowth 21st Project growth standard respectively. Among females, at 36 weeks the mean weight was 1569 g (SD = 201) with a mean weight Z -score of − 2.21 (SD = 1.02) and − 1.95 (SD = 1.36) as per Fenton Growth Chart and Intergrowth 21st Project growth standard respectively. An independent samples t-test showed a significant difference in mean weight Z-scores between birth and 36 weeks postmenstrual age, *p* < 0.001. This was true for both reference growth charts. Therefore, infants were significantly lighter for postmenstrual age at 36 weeks compared to the time of birth. Table 4 shows how growth parameters varied across different birth weight categories.

**Table 3:** Birth weight Z-score and endpoint outcomes at 36 weeks according to Fenton Growth Chart and Intergrowth 21st Project standard. *P* values expressing the difference between these charts are shown. Chi squared test used for the variables “small for gestational age” and “weight above 10^th^ centile at 36 weeks”. Student t-test used for the variables “Delta Z-score”, “Birthweight Z-score” and “Weight Z-score at 36 weeks”

	Number of infants plotted	Fenton Growth Chart	Intergrowth 21st Project	*P* value
Small for gestational age, n (%)	89	13 (14.6%)	27 (30.3%)	0.012
Birth weight Z-score, mean (SD)	89	−0.45 (1.01)	− 0.72 (1.20)	0.107
Weight Z-score at 36 weeks, mean (SD)	89	− 2.50 (1.13)	−2.57 (1.72)	0.725
Delta Z-score, mean (SD)	89	−2.05 (0.87)	−1.87 (1.30)	0.250
Weight above 10th centile at 36 weeks, n (%)	89	11 (12.4%)	20 (22.5%)	0.075

**Table 4:** Birth weight categories and corresponding growth parameters. Weight velocity was calculated from the day birth weight was regained to 36 weeks postmenstrual age

Birth weight	Total number of infants	Infants with good growth (N/%)	Infants with discharge weight > 10th centile (N/%)	Median Gestational age (min - max)	Mean Delta Z-score from birth to 36 weeks (SD)	Mean number of days to regain birth weight (SD)	Mean weight velocity (SD)
600–699	3	0 (0%)	0 (0%)	23 (23–28)	−2.8 (0.7)	10.6 (9.2)	11.6 (2.0)
700–799	13	6 (46.2%)	2 (15.4%)	27 (24–29)	−2.1 (0.9)	14.7 (7.4)	14.0 (3.6)
800–899	35	12 (34.3%)	4 (11.4%)	28 (25–34)	−2.0 (0.8)	19.7 (9.4)	14.0 (3.3)
900–999	41	7 (17%)	5 (12.2%)	28 (23–32)	−2.2 (1.3)	18.5 (11.3)	13.0(2.8)

Table 3 shows that a statistically significant increase in infants labeled as small for gestational age was observed with the Intergrowth 21st Project growth standard compared to the Fenton Growth Chart. There was a larger number of infants who plotted above the 10th centile on the Intergrowth 21st Project standards compared to the Fenton Growth Chart, but this was not statistically significant. There was no statistically significant difference between the Z-scores derived from Fenton Growth Chart and Intergrowth 21st standard for birth weight, weight at 36 weeks and delta Z score.

**Fig. 1:**
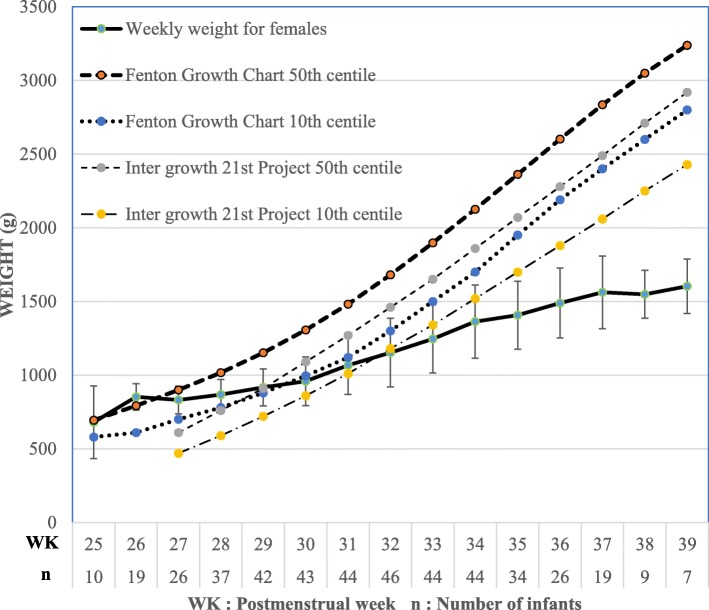
Growth curve for females with standard deviation indicated for each mean weekly weight. Fenton Growth Chart and Intergrowth 21st Project centile lines shown as reference

**Fig. 2:**
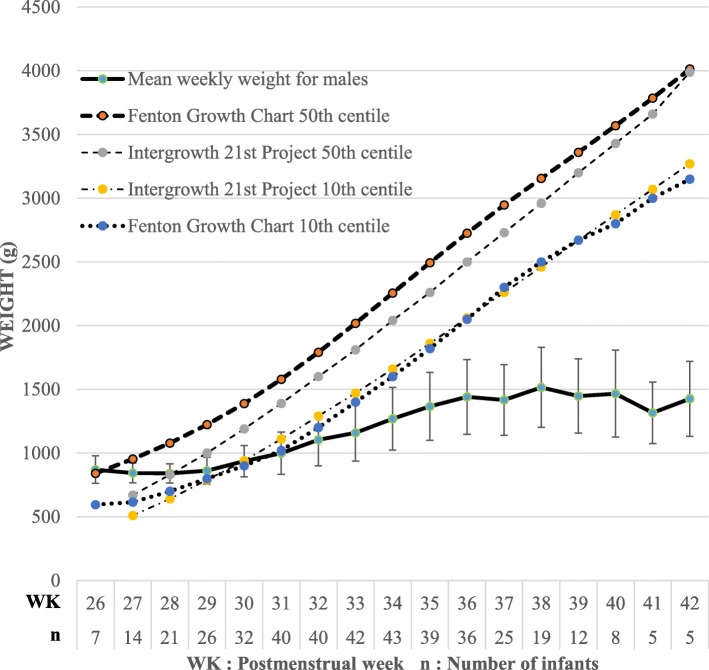
Growth curve for males with standard deviation indicated for each mean weekly weight. Fenton Growth Chart and Intergrowth 21st centile lines shown as reference

Figures 1 and 2 shows how the mean weekly weight for females and males respectively markedly differed from both the Fenton Growth Chart and Intergrowth 21st Project growth standards. Many infants who regained birth weight in 21 days or less did not have an adequate weight velocity afterwards and the vice versa is true as shown in Table 5. Only 25 infants (27.2%) had good growth (regained their birth weight in 21 days or less and subsequently had a weight velocity above 15 g/kg/day) (Table 5).

**Table 5:** Distribution of growth parameters (number of days to regain birth weight and weight velocity) among infants and the resultant discharge weight centile

	Weight velocity > 15 g/kg/day, regained birth weight in 21 days or less	Weight velocity > 15 g/kg/day, regained birth weight after 21 days	Weight velocity < 15 g/kg/day, regained birth weight in 21 days or less	Weight velocity < 15 g/kg/day, regained birth weight after 21 days
Number of infants	25	6	34	27
Infants with weight > Fenton Growth Chart 10th centile at 36 weeks, N (%)	6 (24%)	2 (33.3%)	3 (8.8)	0 (0%)
Infants with weight > Intergrowth 21st Project 10th centile at 36 weeks, N (%)	10 (40%)	3 (50%)	6 (17.6%)	1 (3.7%)
Mean weight velocity, g/kg/day (SD)	16.6 (1.4)	17.5 (2.1)	12.1 (1.9)	11.5 (2.6)
Mean number of days to regain birth weight, days (SD)	14.6 (5.0)	24.3 (2.1)	10.8 (7.0)	29.4 (7.1)

### Macronutrient intake during the first 28 days if life

The mean number of days to reach full enteral feeds was 15.9 days (SD = 6.3) and by the seventh day of life, enteral feeds contributed more than 50% of caloric intake. A plateau of 90–92% mean enteral caloric contribution was reached from the twentieth day. During the first 28 days of life, the mean number of days in which infants received at least 160mls/kg/day of enteral feeds was 12.1 days (SD = 6.3). Thirty-four infants had an adequate caloric intake (above 110 kcal/kg/day). Mean caloric intake during the first 28 days was 97.0 kcal/kg/day (SD = 14.4). A mean caloric intake above 80 kcal/kg/day was reached on day 8, by day 14 the mean caloric intake was above 110 kcal/kg. There was a steady increase in mean weekly caloric intake from 65,7 kcal/kg/day (SD = 18.7) in the first, 101 kcal/kg/day (SD = 8.6) in the second, 118 kcal/kg/day (SD = 5.0) in the third to 123 kcal/kg/day (SD = 2.9) in the fourth week. The mean protein intake was 2.5 g/kg/day (SD = 0.7) with a mean daily intake above 3.0 g/kg/day being reached on the twentieth day of life. The mean lipid intake was 4.4 g/kg/day (SD = 1.1) with a mean daily intake above 5 g/kg/day being reached at the fourteenth day of life.

Pearson correlational analysis showed that during the first 28 days of life, weight velocity increased with a shorter duration to attain full feeds (*r* = −0.38) and the longer the infant remained on feeds of at least 160mls/kg/day (*r* = 0.44). Increases in protein (*r* = 0.38), lipid (*r* = 0.42) and caloric intake (*r* = 0.38) were associated with an increase in weight velocity during the first 28 days of life.

### Complications of prematurity

A comparison was done between the group classified as “good growth” (*n* = 25) and that classified as “poor growth” (*n* = 67). Complications of prematurity which showed a significant association with poor growth were CPAP for more than 2 days, invasive ventilation, oxygen on day 28 of life, late-onset sepsis, PDA and being kept nil per os (excluding the first day of life) (Table 6). No single complication of prematurity was independently associated with neither a weight above the 10th centile at 36 weeks nor a significant difference of delta Z-score between birth and 36 weeks when the Fenton Growth Chart was used. However, use of the Intergrowth 21st Project growth standards showed a significant difference in delta Z-score between infants who were receiving oxygen by day 28 (*p* = 0.024), those with PDA (*p* = 0.001) and sepsis after day 3 (*p* = 0.050) compared to those who did not have such complications. Being kept NPO (*p* = 0.123), receiving for CPAP more than 2 days (*p* = 0.069), antenatal steroids (*p* = 0.219) and invasive ventilation (*p* = 0.254) did not show a significant difference in delta Z-scores compared to those who did not have such complications. A Chi squared test showed a significant association between infants having a weight at 36 weeks less than the 10th centile and those who had sepsis after day 3 of life when the Intergrowth 21st Project growth standards were used (*p* = 0.021).

**Table 6:** Neonatal characteristics associated with poor growth. Univariate analysis with Chi-squared test or Fischer’s exact used as appropriate

Variable		Poor growth	Good growth	n	*P*-value
PDA	Yes	13(100%)	0(0%)	13	0.017
	No	54(68.4%)	25(31.6%)	79	
Sepsis after day 3 of life	Yes	45(84.9%)	8(15.1%)	53	0.002
	No	22(56.4%)	17(43.6%)	39	
Invasive ventilation	Yes	16(94.1%)	1(5.9%)	17	0.034
	No	51(68.0%)	24(32.0%)	75	
CPAP more than 2 days	Yes	26(89.7%)	3(10.3%)	29	0.014
	No	41(65.1%)	22(34.9%)	63	
Nil per os (excluding the first day of life)	Yes	49(80.3%)	12 (19.7%)	61	0.023
	No	18(58.1%)	1341.9%)	31	
Oxygen on day 28 of life	Yes	54(79.4%)	14(20.6%)	68	0.017
	No	13(54.2%)	11(45.8%)	24	

## Discussion

This study details the pathological growth pattern in ELBW infants leading to an undesirable weight at 36 weeks postmenstrual age. In the present study, infants regained birth weight in 18.3 days and had a mean weight velocity of 13.5 g/kg/day. Although a recommendation on the minimum acceptable weight velocity does not exist [24], the mean weight velocity in the present study was comparable to previous studies [11, 12] and approaches the frequently used minimum of 15 g/kg/day [24]. The present study shows that many infants who regained birth weight in 21 days or less did not have an adequate weight velocity afterwards and vice versa. This resulted in failure of most infants to attain a weight above the 10th centile at 36 weeks on both the Fenton Growth Chart and Intergrowth 21st Project Postnatal Chart. Findings by Dejhalla et al. among 21 ELBW infants with an uncomplicated hospital stay showed much better outcomes with 71% of infants having a weight above the 10th centile on discharge [25]. The study excluded infants with blood culture proven sepsis, necrotizing enterocolitis and neonatal surgery among other complications. This highlights the importance of identifying the neonatal characteristics associated with growth.

The mean caloric intake in the present study is comparable to previous studies but lags in protein intake [6, 25]. Indeed, during the first few days of life, infants in the present study had a more rapid advancement of caloric intake and enteral feeds compared to the study by Dejhalla et al. whose infants had a better outcome. The determining factor could have been the protein intake, in the study by Dejhalla et al., a protein intake of 3.0 g/kg/day was reached by the tenth day compared to the twentieth day in the present study.

CPAP duration exceeding 2 days, invasive ventilation, oxygen on day 28, late-onset sepsis, being kept nil per os and PDA were associated with poor growth (number of days to regain birth weight and weight velocity). In a South African study by Mudahemuka et al., the growth of 69 VLBW infants who survived to discharge from the neonatal unit was reviewed. In the study, a growth velocity < 14 g/kg/day was associated with antenatal steroids and the number of days nil per os without ITN correlated negatively with the discharge weight Z-score [11]. In the present study, no association was demonstrated between growth and antenatal steroids, however the definition of good growth velocity was different from that use by Mudahemuka et al. Alejandro et al. demonstrated multiple neonatal characteristics associated with a significant decline in Z-score from birth to discharge including mechanical ventilation and PDA among 130 infants with a birth weight less than 1500 g [26]. These findings approximate the results of the present study, however, the use of discharge weight Z-score as an endpoint by Alejandro et al. presents a possibly significant difference in study design.

In the present study, a greater portion of infants were considered small for gestation age using the Intergrowth 21st Project growth standards compared to the Fenton Growth Chart. The increase in number of infants plotting above the 10th centile at 36 weeks using the Intergrowth 21st standards approaches statistical significance. These findings are consistent with a study conducted by Funda et al. on 248 infants in Turkey comparing the Fenton Growth Chart to Intergrowth 21st Project standards [27]. Funda et al. did not find any increased risk for early morbidity in the new small for gestational age infants identified by the Intergrowth 21st Project growth standards. However, in the present study, although no neonatal factors were associated with a weight > 10th centile at 36 weeks when the Fenton Growth Chart was used, this was not the case with the Intergrowth 21st Project growth standards. When Intergrowth 21st Project growth standards were used, the decline in Z-score from birth to 36 weeks was significantly larger among infants with PDA, sepsis after day 3 of life and those who remained on oxygen at day 28 of life. This suggests that Intergrowth 21st Project growth standards may provide a better association between growth and neonatal complications in our population compared to the traditionally used Fenton Growth Chart. This finding reverberates the concern that intrauterine growth may not be the appropriate standard for extrauterine growth in premature infants, furthermore, whether the Fenton Growth Chart is suitable for our population [17].

## Study limitations

This was a retrospective study and infants were managed as per attending physicians’ discretion. The method by which gestational age was determined was not standardized. A larger sample would have allowed a better description of rare complications of prematurity and infants who attended follow-up clinic at 12 months corrected age as only a few infants were picked up. Thirty-six infants (40.4%) were discharged before 36 weeks had their discharge weight Z-score analyzed as the one at 36 weeks, however, the resulting error was assumed to be small since the mean postmenstrual age at discharge was 36.7 weeks. Infants discharged before 36 weeks had a mean postmenstrual age of 34.5 weeks (SD = 1.1) at the time of discharge.

However, this study provides a detailed description of macronutrient intake, such detail has rarely been described in South Africa and other middle and low-income countries. The present study also explores the usefulness of the Intergrowth 21st Project growth standards compared with the traditionally used Fenton Growth Charts.

## Conclusion and recommendations

The growth of ELBW infants at CMJAH is suboptimum. Multiple complications of prematurity are associated with poor growth and nutritional intake correlates positively with weight velocity. There is need for a multifaceted approach to address characteristics associated with poor growth. Respiratory complications of prematurity can be reduced by increased coverage of antenatal steroids. Infants are likely receiving NCPAP after a significant delay at CMJAH neonatal unit, there is a need to avail enough NCPAP units in the Transitional Unit. A protocol addressing both volume and nutritional requirements during initiation of enteral feeds is needed. Consideration should be given to start ITN by 24 h rather than the current 48 h in infants not on full enteral feeds. Small enteral feeds can also be introduced on the first day of life. Chorioamnionitis is likely underreported, more vigilant surveillance and treatment in pregnant mothers could prevent premature delivery. Strong consideration should be given for the use of Intergrowth 21st Project growth standards as reference for normal postnatal growth in premature infants instead of the Fenton Growth Chart currently in use. Unlike the Fentons Growth Charts, use of the Intergrowth 21st Project growth standards revealed the association between neonatal factors and poor growth. A large multicenter prospective study is needed to assess the extent to which changes brought about by the Intergrowth 21st Project growth standards correlate with long term outcomes.

## Data Availability

The datasets used during the current study are available from the corresponding author on reasonable request.

## Abbreviations

CMJAH: Charlotte Maxeke Johannesburg Academic Hospital
CPAP: Continuous positive airway pressure
ELBW: Extremely low birth weight (birth weight less than 1000 g)
ITN: Intravenous total nutrition
NEC: Necrotising enterocolitis
RDS: Respiratory distress syndrome
REDCAP: Research electronic data capture
ROP: Retinopathy of prematurity
VLBW: Very low birth weight (birth weight less than 1500 g)
